# Comprehensive Investigation on the Influence of Dietary Soybean Oil Levels in a Soybean Oil‐Based Diet on Gibel Carp (*Carassius gibelio*): Insights Regarding Growth, Nutrition, and Physiological, Metabolic, and Transcriptomic Analyses

**DOI:** 10.1155/anu/9199711

**Published:** 2026-09-29

**Authors:** Xiangning Chen, Huiqing Li, Letian Qu, Zheqiu Li, Zhiwen Chen, Zikui Yang, Teng Xia, Jianhe Xu, Hanliang Cheng, Qingqing Shi

**Affiliations:** ^1^ Jiangsu Key Laboratory of Marine Biotechnology Jiangsu Ocean University Lianyungang 222005 China hhit.edu.cn; ^2^ Jiangsu Key Laboratory of Marine Bioresources and Environment Jiangsu Ocean University Lianyungang 222005 China hhit.edu.cn; ^3^ Department of Hematology Laboratory Northern Jiangsu People’s Hospital Affiliated to Yangzhou University Yangzhou University Yangzhou 225001 China yzu.edu.cn

**Keywords:** *Carassius gibelio*, growth and nutritional performance, lipid levels of soybean oil-based diets, lipid metabolism-associated phenotypes, transcriptome analysis

## Abstract

Five isonitrogenous (35%) diets with different lipid contents were prepared by adding 0%, 3%, 6%, 9%, and 12% soybean oil (SO) as the single lipid source (with groups designated SO0, SO3, SO6, SO9, and SO12, respectively), followed by a 12‐week feeding experiment in juvenile gibel carp (*Carassius gibelio*) (initial weight: 11.76 ± 0.09 g). The final weight (FW), weight gain rate (WGR), and specific growth rate (SGR) were significantly higher in gibel carp fed diets containing 6%–12% SO than those of others (*p*  < 0.05), while the feed conversion ratio (FCR) was significantly reduced in the SO6 group (*p*  < 0.05). Muscle crude fat and polyunsaturated fatty acid (PUFA) levels significantly increased as the dietary SO content increased (*p* < 0.05). Meanwhile, muscle crude protein was present in notably higher amounts in fish receiving 6%–9% SO (*p* < 0.05). Serum low‐density lipoprotein (LDL), triglyceride (TG), and total cholesterol (T‐CHO) continuously increased with increasing SO levels (*p* < 0.05). Histopathological abnormalities were observed in the local regions of the hepatopancreas after supplementation with 9%–12% SO. Most genes involved in lipid metabolism were upregulated to varying degrees (*p* < 0.05) as dietary SO content increased, especially lipogenic genes. About 4481 differentially expressed genes (DEGs) were screened in the hepatopancreas between the SO12 and SO0 group, primarily linking to metabolism‐related pathways, including steroid biosynthesis. Conclusively, the available content of SO as the sole fat source in diets for juvenile gibel carp was found to be 6%–9%, within which the optimal SO inclusion was 6.37% according to FCR–based regression analysis. Excessive supplementation of dietary SO (12% or beyond) could expedite hepatic lipid metabolism, particularly lipogenesis, by stimulating steroidogenesis, thereby evoking fat over‐accumulation, lipid metabolism disorders, and other adverse effects in gibel carp. These results provide synthetic data for better understanding the advantages, disadvantages, and relevant mechanisms of SO–based feed for gibel carp and for optimizing SO–based aquafeeds in farmed fish.

## 1. Introduction

In the face of the high costs, restricted yields, and potential environmental challenges related to high‐quality protein sources (especially fish meal) [1, 2], combined with the low utilization of dietary carbohydrates in fish [3], dietary lipids have become crucial in aquaculture. Fat and the relevant components of dietary lipids, such as phospholipids and sterols, not only provide essential fatty acids, energy substrates, and spare dietary protein, but also participate in physiological processes in fish; both of these factors influence fish growth, health, and flesh quality [4–6]. Fish oil (FO) is a well‐recognized optimal lipid for aquafeeds, distinguished by an abundance of n‐3 polyunsaturated fatty acids (PUFAs) and a balanced FA composition [7, 8]. However, the limited output, skyrocketing price, and easy oxidation and deterioration of FO due to the continuous increase in aquaculture have driven a switch toward plant oils to diminish the need for FO aquafeeds [7, 9, 10].

Soybean oil (SO) is a typical plant oil and is extensively used in aquafeeds due to its wide availability and high n‐6 PUFAs content [7]. No unfavorable growth performance, immune response, or antioxidant effects have been reported for multiple fishes (especially freshwater species) [7, 11–13] after the full replacement of FO with SO. Nevertheless, excessive n‐6 FA intake is hazardous to marine fish health, causing attenuated immunity, increased inflammatory and oxidative stress, weakened disease resistance, etc. [9, 10, 14]. Marine fishes’ lower ability to synthesize n‐3 PUFAs (especially n‐3 long‐chain PUFAs) from n‐6 FAs compared to freshwater fish [15, 16] can trigger this phenomenon. It is also attributed to an imbalance in the n‐3/n‐6 PUFA ratio [10] or relatively low dietary n‐3 PUFA contents; both are caused by SO and other vegetable oils containing high doses of n‐6 FAs. Therefore, the application of SO in aquafeeds, particularly as the sole dietary fat source, needs to be adequately evaluated to achieve a balanced FA profile, the pro‐growth and protein‐sparing effect, and health and nutrition risks should be eliminated/minimized.

Gibel carp (*Carassius gibelio*), belonging to the genus Carassius within the family Cyprinidae, is an economically important aquaculture species in China, with advantages including rapid growth, high production, strong disease resistance and adaptability, and delicious flesh [4, 17, 18]. Diets formulated with a single vegetable oil, such as SO, have been demonstrated to be applicable in multiple studies, including on some cyprinid fishes [7, 10, 19, 20]. However, dietary lipid requirement and the corresponding lipid metabolism response vary among fish species, life stages, and aquatic environments [7, 21], and even for different varieties of the same species [22]. Using SO as a sole fat source, the suitable dietary fat content of large‐sized common carp (*Cyprinus carpio*) ranged from 59.5 to 70.4 g/kg [19], whereas the recommended dietary lipid level for grass carp (*Ctenopharyngodon idella*) was 8%–11% [7]. Furthermore, the common tendency to employ a higher content of dietary lipids in aquafeeds has generated controversy, with drawbacks including growth hindrance, lower feed intake, abnormal hepatic lipid deposition, and poor nutritional value [19, 23–25]. Therefore, for any given fat source, it is essential to identify the appropriate amount for farmed fish. To date, comprehensive studies on the application of SO as a single fat source in gibel carp feeds are still limited, warranting further exploration.

Based on the aforementioned findings, diets featuring SO as the sole lipid source were formulated and implemented to systematically assess how SO–based diets with different lipid contents affect the growth, flesh nutrition, physio–biochemical indicators, molecular responses, and transcriptome characteristics related to hepatic lipid metabolism in juvenile gibel carp “CAS III,” a widely promoted variety of gibel carp. This research can not only help optimize the SO contents in SO–based formulations for gibel carp and promote further employment of SO‐ and other plant oil‐based diets in aquatic animals but also facilitate an intensive elaboration of the underlying mechanism of SO–based feeds on lipid metabolism in fish.

## 2. Materials and Methods

### 2.1. Ethics Statement

This study was approved by the Animal Care and Use Committee of Jiangsu Ocean University (Approval No. 2020‐37). All experimental manipulations involving fish were performed in accordance with the guidelines for the care and use of laboratory animals in China.

### 2.2. Experimental Feed Formulation and Feeding Trials

Following a previous study [7], five isonitrogenous diets (with about 35% crude protein) were designed with different lipid contents using SO as the sole lipid source and fishmeal, soybean meal, canola meal, and cottonseed meal as the main protein sources. These diets were supplemented with 0%, 3%, 6%, 9%, and 12% SO—referred to as SO0, SO3, SO6, SO9, and SO12, respectively—and were sealed and stored at 4°C until the feeding trial. The formulation and components of the experimental diets are detailed in Supporting Information 1: Table S1, while fatty acid compositions are presented in Table 1 and Supporting Information 1: Table S2. Diets were prepared as previously described [7].

**Table 1:** Fatty acid contents of the experimental feeds (mg/g, per diet).

Items	Groups				
	SO0	SO3	SO6	SO9	SO12
C12:0	0.03 ± 0.00^c^	0.03 ± 0.00^c^	0.04 ± 0.00^b^	0.04 ± 0.00^b^	0.06 ± 0.00^a^
C14:0	1.65 ± 0.08^d^	1.99 ± 0.16^c^	2.66 ± 0.15^b^	2.84 ± 0.12^b^	3.08 ± 0.15^a^
C16:0	8.67 ± 0.40^e^	17.50 ± 0.83^d^	24.57 ± 1.48^c^	32.21 ± 0.98^b^	40.36 ± 1.01^a^
C18:0	1.82 ± 0.08^e^	4.73 ± 0.11^d^	7.72 ± 0.56^c^	10.24 ± 0.13^b^	12.98 ± 0.28^a^
C20:0	–	0.31 ± 0.01^d^	0.59 ± 0.08^c^	0.69 ± 0.02^b^	0.79 ± 0.01^a^
∑SFA	12.18 ± 0.55^e^	24.56 ± 0.84^d^	35.57 ± 1.59^c^	46.02 ± 1.00^b^	57.27 ± 1.07^a^
C16:1	0.57 ± 0.03^e^	0.74 ± 0.01^d^	0.93 ± 0.06^c^	1.00 ± 0.02^b^	1.09 ± 0.02^a^
C18:1n–9	2.71 ± 0.12^e^	6.69 ± 0.25^d^	11.47 ± 0.41^c^	16.35 ± 0.48^b^	21.52 ± 0.25^a^
C20:1n–9	0.33 ± 0.02^a^	0.09 ± 0.00^c^	0.13 ± 0.00^b^	0.01 ± 0.00^d^	0.01 ± 0.00^d^
∑MUFA	3.61 ± 0.17^e^	7.52 ± 0.27^d^	12.53 ± 0.42^c^	17.36 ± 0.49^b^	22.62 ± 0.25^a^
C18:3n–3(ALA)	0.65 ± 0.03^e^	2.00 ± 0.18^d^	4.41 ± 0.68^c^	6.21 ± 0.17^b^	8.57 ± 0.28^a^
C20:5n–3(EPA)	0.79 ± 0.04^d^	1.00 ± 0.01^c^	1.22 ± 0.04^b^	1.29 ± 0.03^b^	1.35 ± 0.02^a^
C22:6n–3(DHA)	0.53 ± 0.03^e^	0.66 ± 0.02^d^	0.83 ± 0.02^b^	0.78 ± 0.00^c^	0.92 ± 0.04^a^
∑n‐3 PUFA	1.97 ± 0.09^e^	3.66 ± 0.19^d^	6.46 ± 0.69^c^	8.28 ± 0.17^b^	10.84 ± 0.29^a^
C18:2n–6(LA)	3.95 ± 0.18^e^	15.29 ± 0.59^d^	25.85 ± 2.03^c^	38.17 ± 1.14^b^	48.49 ± 0.51^a^
C20:4n–6(ARA)	–	0.06 ± 0.00^c^	0.08 ± 0.00^a^	0.07 ± 0.00^b^	0.07 ± 0.00^b^
∑n‐6 PUFA	3.95 ± 0.18^e^	15.36 ± 0.60^d^	25.93 ± 2.03^c^	38.25 ± 1.14^b^	48.58 ± 0.51^a^

*Note:* Experimental data are presented as mean ± standard deviations (SD) (*n* = 3). Unlike lowercase superscript letters in the same row denote statistical significance among groups (*p* < 0.05). (–) not detected in this study.

Abbreviations: ALA, *α*‐linolenic acid; ARA, arachidonic acid; DHA, docosahexenoic acid; EPA, eicosapentaenoic acid; LA, linoleic acid; MUFA, monounsaturated fatty acids; n‐3 PUFA, omega 3 polyunsaturated fatty acids; n‐6 PUFA, omega 6 polyunsaturated fatty acids; SFA, saturated fatty acids.

Healthy juvenile gibel carp (*Carassius gibelio* var. CAS III) were purchased from Huandun fishery (Lianyungang, China) and cultured in the indoor flow‐through system located in Jiangsu Key Laboratory of Marine Biotechnology, Jiangsu Ocean University (Lianyungang, China) for a 2‐week acclimation period. Juveniles were fed the SO0 diet (i.e., the control diet supplemented with 0% SO) to satiation four times per day (8:00, 11:00, 14:00, and 17:00) in the acclimation phase.

At the initiation of the feeding study, 450 gibel carp (body weight: 11.74 ± 0.08 g) were randomly distributed into 15 tanks with 240 L (i.e., 30 fish/tank, 3 replicates/group, 5 groups in total). Experimental fish were hand‐fed the corresponding diets four times daily to full satiation for 12 weeks. Throughout acclimation and the feeding trial, diet residue and feces were collected by siphoning within 60 min of feeding. The water environment of the flow‐through system was maintained as follows: water temperature, 20–25°C; dissolved oxygen >5 mg/L; pH 7–8; ammonia nitrogen <0.14 mg/L; and photoperiod12 h/12 h light/dark cycle.

### 2.3. Sampling

Once the 12‐week feeding trial was over, experimental fish underwent 24 h fasting and then the fish number and total weight were recorded. Nine gibel carp in each tank were randomly netted, fully anesthetized under 20 mg/L MS‐222 (Merck KgaA, Darmstadt, Germany), and individually measured for body weight and body length. Blood was acquired from the tail vein of each fish and immediately centrifuged (3000 rpm, 4°C, 10 min) to harvest the supernatant, which was cryopreserved at − 80°C until serum detection. Thereafter, fish were mechanically dissected on ice to separate visceral tissues, and the skinless muscle samples were isolated from the left‐dorsal side, flash‐frozen, and kept at −80°C for nutrition composition analysis. Similarly, the freshly isolated hepatopancreas was rapidly placed in liquid nitrogen and transferred to −80°C for RNA‐seq and gene expression assay. Another three individuals from each tank were randomly selected and dissected on ice to separate the hepatopancreas. These isolated hepatopancreases were immediately fixed with 4% paraformaldehyde (PFA) for histological examination.

### 2.4. Growth Parameter Calculation

The total number and final weight (FW) of the experimental fish in each tank were counted to calculate the following growth indicators: weight gain rate (WGR), specific growth rate (SGR), survival rate (SR), feed conversion ratio (FCR), viscerosomatic index (VSI), hepatosomatic index (HSI), and condition factor (CF). The corresponding equations of the above‐mentioned parameters are referred to in previous studies [7, 26].

### 2.5. Proximate, Fatty Acid, and Amino Acid (AA) Composition Analysis

Three muscle samples were clipped from the dorsal sides of three gibel carp in each tank, mixed as one representative sample, and evaluated in the relevant assays of nutritional composition. Proximal compositions of fish muscle were measured with reference to the standard protocols [27]. Specifically, moisture content was estimated by drying the muscle specimens to constant mass at 105°C. Crude protein was detected on a FOSS Kjeltec 8400 analyzer (FOSS, Denmark) following the Kjeldahl method. Using SoxtecTM‐8000 Extraction (FOSS, Denmark), the ether extraction method with petroleum ether (boiling range in 30–60°C) as a solvent was applied to determine the crude fat content. Crude ash was quantified by incinerating the muscular sample in a muffle furnace (550°C, 4–6 h).

Total lipid of the fish muscle was extracted via the chloroform–methanol method as previously described [28]. Fatty acid was converted using methyl esterification and then detected using the gas chromatography–mass spectrometry (GCMS) method on a GCMS‐QP2010 SE GCMS system (Shimadzu Scientific Instruments Pty Ltd., Kyoto, Japan) with a 60 m × 0.15 mm column (DB‐23, Agilent Technologies, CA, USA). The identification and quantification of fatty acids were performed based on the peak area normalization method. The fatty acid profile of each sample was represented as the percentage of each fatty acid relative to total fatty acids. Detailed information on the procedures and parameters of fatty acid analysis can be found in our previous publication [7].

The oxidative hydrolysis method using peroxyformic acid and hydrochloric acid was adopted to digest muscle protein and release AAs. The digestion solution was deoxygenated by flushing with nitrogen, heated at 110°C for 22–24 h, and concentrated in vacuo. After redissolution in hydrochloric acid, the obtained sample solution was filtered into a sample vial using a 0.22 μm filter membrane (Slgvr33rs, Millipore, Germany). The composition and content of AAs in the sample was measured on a Hitachi L‐8900 AA analyzer (Hitachi, Japan), following the experimental parameters in the previous methodology [28].

### 2.6. Analysis of Serum Biochemical Indicators

Biochemical indicators in serum, including alanine aminotransferase (ALT), aspartate transaminase (AST), high‐density lipoprotein (HDL), low‐density lipoprotein (LDL), total TG, and total CHO, were quantified using the respective assay kits (Nanjing Jiancheng, Nanjing, China). The experimental manipulations were run on a Multiskan FC photometer (Thermo Scientific, Waltham, MA, USA) following the standard protocol suggested by the corresponding kits.

### 2.7. Hepatopancreatic Histological Observation

For microstructural analysis, paraffin sections of hepatopancreatic samples were obtained and stained using the same manipulations as described in our previous study [4]. HE‐stained hepatopancreatic specimens were observed and photomicrographed under the light microscope (Eclipse Ci‐L, Nikon, Tokyo, Japan). The vacuole area percentage of HE‐stained hepatopancreas was calculated using the ImageJ software (National Institutes of Health, Bethesda, MD, USA).

### 2.8. Library Preparation, Sequencing, and Transcriptome Analysis

Representative hepatopancreases from the SO0 and SO12 groups were selected for transcriptome sequencing due to obvious differences in growth, nutrition, and lipid‐metabolism‐associated phenotypes between gibel carp receiving 0% and 12% SO. The total RNA of each hepatopancreas specimen (i.e., three hepatopancreases per tank mixed as one sample and two dietary lipid levels X three biological replicates; six samples in total) was extracted via the Trizol–chloroform method and then subjected to qualitative and quantitative evaluation using gel electrophoresis and an Agilent 2100 Bioanalyzer (Agilent Technologies, CA, USA), respectively. Only hepatopancreatic RNA with a high purity (OD_260/280_: 1.8–2.10) and a high RIN value (>7.8) was used for the library generation and sequencing analysis, which were performed on an Illumina sequencing system (HiSeq TM 2500) by Sangon Biotech Co., Ltd. (Shanghai, China). Following a previously published methodology [4], raw sequencing data were processed for quality checks and filtered, aligned, and assembled. All original RNA‐seq data have been deposited in a sequence read archive (SRA) database with the accession no. PRJNA1404241.

The DEGseq R package was applied to screen differentially expressed genes (DEGs) between groups and the corresponding results were analyzed. The default threshold for identifying DEGs included |log2 (fold change)| > 1 and an adjusted *p*‐value <0.05. The identified DEGs were subjected to gene ontology (GO) enrichment analysis by topGO R package. The Kyoto Encyclopedia of Genes and Genomes (KEGG) pathway enrichment analysis was implemented and visualized by the clusterProfiler software. A *p*‐value <0.05 was set as the statistical threshold for the significantly enriched GO term and KEGG pathway.

### 2.9. Quantitative Real‐time Polymerase Chain Reaction (qRT‐PCR) Assay

Total RNA of the hepatopancreas samples (hepatopancreases from three fish individuals per tank mixed as one sample) was extracted according to the instructions of an RNAprep Pure Tissue Kit (DP431, Tiangen, Beijing, China). After checking for concentration and integrity, the isolated RNA (1 μg per sample) was subjected to reverse transcription with a FastKing gDNA Dispelling RT SuperMix kit (KR118, Tiangen, Beijing, China). qRT‐PCR reactions were carried out on the StepOne Plus Real‐Time PCR system (Applied Biosystems, CA, USA) using the TB Green Premix Ex Taq II kit (RR820A, TaKaRa, Dalian, China). A qRT‐PCR assay of lipid metabolism‐associated genes and selected DEGs was run following the procedures reported earlier [7]. Elongation factor 1 *α* (*ef1a*) and ribosomal protein L13a (*rpl13a*) served as the reference genes based on their stable expressions in this study. The relative abundances of target genes were calculated using the 2^−ΔΔCt^ method. Sequence information on specific primers is presented in Table 2 and Supporting Information 1: Table S3.

**Table 2:** Nucleotide sequences of primers used for qRT‐PCR assay.

Gene	Forward primer (5′− 3′)	Reverse primer (5′− 3′)	Amplification efficiency	Length (bp)	Accession number
*acsl1*	GGGAGAGGTGTGTGTGAAAGGA	CAGTGTGCAGCCAGCCGTC	0.96	101	XM_052546481
*adipor2*	CGAAAAGGAGGAGAAAACA	CTTCAGCCAATCAGGGAG	1.03	172	XM_052581973
*ampkβ1*	TGGAGCTCGACCCAAAATCC	AACACAGTGGGCCTTTCCTC	1.01	141	XM_051904021
*cd36*	GCAGAACGCTGACGATGTTG	TAGGCTCAAAGATGGCACCC	0.93	161	XM_052577052
*cpt1a*	TCGAGTTCCCGGTGTAGAAA	ACTCTGTCACCCGCTGTTAAG	0.97	222	XM_052597800
*elovl4*	GACGCTCTTGAATCCCGAA	CTGAGCCCTAGCTTAAACACC	0.92	187	XM_052541198
*elovl6*	TAAGTGCGCTAGTCTACCA	TACATGAGTGACGCCCACA	0.98	82	XM_052571797
*elovl7*	GCTGCTTATGGCCACTCCAT	ACTGCTATAATCCACGAGGTCA	0.93	235	XM_052568089
*fabp*	CCTTTGACTATTCTCTGGC	TTTCCGTTGTCCTTGC	0.94	100	XM_052546129
*fad*	TCTGCCTTACAACCATCAGC	CTTATACACCACGCAAGGTC	0.91	135	XM_051883101
*lpl*	AGTACGCAGATGCCCAAAG	CTGGCCTCTGAATCCCAATAC	1.02	111	XM_051909470
*pla2*	TTGTAAAAGAGATCGACGAAC	TAACCTGCTTCATGTGGAC	0.90	151	XM_052557018
*ef1a*	CGCCAGTGTTGCCTTCGT	CGCTCAATCTTCCATCCCTT	0.95	99	XM_052583620
*rpl13a*	CTTCTGGAGGACAGTAAGAGGTATGC	GGAGGAGGGATGCCATCAAAGAC	0.91	96	XM_052580684

*Note*: Primers sequences for qRT‐PCR assay were designed using the Primer Premier 5 or obtained by previous studies [4, 7].

Abbreviations: *acsl1*, long‐chain acyl‐CoA synthases 1; *adipor2*, adiponectin receptor 2; *ampkβ1*, AMP‐activated protein kinase beta 1; *cd36*, fatty acid translocase/CD36; *cpt1a*, carnitine palmitoyltransferase 1 alpha; *ef1a*, elongation factor 1 alpha; *elovl4*, elongase of very long‐chain fatty acids 4; *elovl6*, elongase of very long‐chain fatty acids 6; *elovl7*, elongase of very long‐chain fatty acids 7; *fabp*, fatty acid binding protein; *fad*, fatty acid desaturases; *lpl*, lipoprotein lipase; *pla2*, phospholipase A2; *rpl13a*, ribosomal protein L13a.

### 2.10. Statistical Analysis

Prior to one‐way analysis of variance (ANOVA), all data were subjected to tests of normality and variance homoscedasticity. Compliant data were further assessed by ANOVA with Tukey’s or Welch’s multiple range tests. For non‐normally distributed data, the Kruskal–Wallis method with Dunn’s post hoc test was employed for multiple comparisons among individual groups. Values of the experiment data were expressed as mean and standard deviations, that is, mean ± SD, and visualized using GraphPad Prism Software (version 9.0, GraphPad, La Jolla, CA, USA). Statistical assessments were conducted via SPSS software (version 23.0, SPSS Inc., Chicago, IL, USA). A value of *P* less than 0.05 was considered statistically different.

## 3. Results

### 3.1. Growth Indicators

The values of FW, VSI, and CF were obviously greater in the other three groups compared with the control group (i.e., SO0 group) (*p* < 0.05; Table 3). The same was true for WGR and SGR (*p* < 0.05; Table 3). Moreover, FW, VSI, and CF exhibited the significantly elevating trends with the increasing lipid content in the SO–based diet and reached their respective peaks in the SO12 group (*p* < 0.05; Table 3). WGR and SGR for groups receiving 6%–12% dietary SO were not statistically different across the three groups (*p* > 0.05; Table 3), but markedly higher than those of the SO3 group (*p* < 0.05; Table 3). When increasing the SO content from 0% to 12%, FCR and HSI initially decreased and then increased, achieving the minimum in the SO6 group (*p* < 0.05; Table 3). The optimal dietary SO addition for gibel carp was 6.37% according to the binary regression analysis for FCR (Supporting Information 2: Figure S1). As for survival rate (SR), no obvious differences were observed in fish among all groups (*p* > 0.05; Table 3).

**Table 3:** Growth parameters of juvenile gibel carp fed SO–based diets with different lipid contents.

Items	Groups				
	SO0	SO3	SO6	SO9	SO12
IW (g)	11.76 ± 0.09	11.89 ± 0.07	11.57 ± 0.13	11.73 ± 0.04	11.75 ± 0.05
FW (g)	31.30 ± 0.80^d^	36.73 ± 0.49^c^	41.85 ± 1.20^b^	42.74 ± 0.46^ab^	44.00 ± 0.99^a^
WGR (%)	166.11 ± 5.67^c^	208.92 ± 4.12^b^	263.02 ± 5.82^a^	264.44 ± 4.80^a^	274.37 ± 0.26^a^
SGR	1.16 ± 0.08^c^	1.31 ± 0.09^b^	1.53 ± 0.06^a^	1.54 ± 0.09^a^	1.57 ± 0.06^a^
SR (%)	98.89 ± 1.92	100 ± 0.00	100 ± 0.00	96.67 ± 3.33	100 ± 0.00
FCR	1.33 ± 0.08^a^	1.23 ± 0.03^ab^	1.18 ± 0.01^b^	1.21 ± 0.10^ab^	1.36 ± 0.13^a^
CF (%)	1.67 ± 0.08^c^	1.74 ± 0.07^bc^	1.80 ± 0.07^ab^	1.80 ± 0.06^ab^	1.84 ± 0.11^a^
HSI (%)	3.41 ± 0.42^ab^	3.08 ± 0.51^ab^	2.74 ± 0.13^b^	3.50 ± 0.54^ab^	3.90 ± 0.36^a^
VSI (%)	10.37 ± 0.41^c^	10.61 ± 0.25^bc^	12.07 ± 0.26^ab^	12.08 ± 0.63^ab^	13.32 ± 0.88^a^

*Note:* Experimental data are expressed as mean ± SD (*n* = 3). Unlike lowercase superscript letters in the same row denote statistical significance among groups (*p* < 0.05).

Abbreviations: CF, condition factor; FCR, feed conversion ratio; FW, final weight; HSI, hepatosomatic index; IW, initial weight; SGR, specific growth rate; SR, survival rate; VSI, viscerosomatic index; WGR, weight gain rate.

### 3.2. Muscle Proximate Composition

The crude fat content in muscle gradually increased and peaked in the SO12 group as dietary SO content increased (*p* < 0.05; Table 4), while the opposite trend was observed in muscle ash (*p* < 0.05; Table 4). Fish in the SO6 and SO9 groups showed higher crude protein contents compared to other groups (*p* < 0.05; Table 4). Only muscle moisture was not significantly altered, regardless of the lipid content in SO–based diets (*p* > 0.05; Table 4).

**Table 4:** Nutritional composition of muscle in juvenile gibel carp fed SO–based diets with different lipid contents.

Items	Groups				
	SO0	SO3	SO6	SO9	SO12
Moisture (%)	77.61 ± 0.07	78.70 ± 0.11	78.21 ± 0.62	77.77 ± 0.50	77.22 ± 1.35
Crude protein (% wet mass)	17.94 ± 0.05^b^	17.93 ± 0.16^b^	18.27 ± 0.60^a^	18.15 ± 0.34^a^	18.07 ± 0.80^ab^
Crude lipid (% wet mass)	1.74 ± 0.02^c^	1.58 ± 0.01^c^	2.71 ± 0.04^b^	2.99 ± 0.08^b^	3.77 ± 0.33^a^
Ash (% wet mass)	1.19 ± 0.02^a^	1.09 ± 0.02^ab^	1.07 ± 0.05^b^	1.08 ± 0.03^ab^	1.05 ± 0.07^b^

*Note:* Experimental data are expressed as mean ± SD (*n* = 3). Unlike lowercase superscript letters in the same row denote statistical significance among groups (*p* < 0.05).

The SFA and MUFA contents in muscle both exhibited constant decline with increasing dietary SO levels (*p* < 0.05; Table 5, Supporting Information 2: Figure S3), even though no marked differences in MUFA were found between the SO 9 and SO 12 groups (*p* > 0.05; Table 5). A similar decreasing trend was observed in the EPA level of the gibel carp muscle (*p* < 0.05; Table 5) in response to an increase in dietary SO supplementation from 0% to 12%. The proportions of DHA and n‐3 PUFA were markedly higher in the muscular samples of the SO9 and SO12 groups in comparison to those of other groups (*p* < 0.05; Table 5). Additionally, the n‐3 PUFA/n‐6 PUFA content was significantly higher in the SO0 group compared to other groups (*p* < 0.05; Table 5), despite no statistical differences in its ratio among the groups receiving 3%–12% dietary SO (*p* > 0.05; Table 5).

**Table 5:** Fatty acid profiles in the muscle of juvenile gibel carp fed SO‐based diets with different lipid contents (total identified fatty acids, %).

Items	Groups				
	SO0	SO3	SO6	SO9	SO12
C12:0	0.07 ± 0.03^a^	0.06 ± 0.00^a^	0.06 ± 0.01^a^	0.04 ± 0.01^b^	0.03 ± 0.01^b^
C14:0	3.80 ± 0.10^a^	3.00 ± 0.12^b^	2.60 ± 0.20^b^	2.09 ± 0.19^c^	1.99 ± 0.05^c^
C16:0	43.48 ± 0.47^a^	39.74 ± 0.14^b^	37.08 ± 0.35^c^	36.64 ± 0.97^d^	32.05 ± 0.42^e^
C18:0	12.97 ± 0.27^a^	12.85 ± 0.24^a^	12.81 ± 0.60^a^	12.18 ± 0.37^b^	11.89 ± 0.16^b^
∑SFA	60.32 ± 0.28^a^	55.65 ± 0.25^b^	52.54 ± 0.78^c^	48.95 ± 1.08^d^	45.97 ± 0.39^e^
C16:1	3.49 ± 0.04^a^	2.00 ± 0.05^b^	1.53 ± 0.12^c^	1.18 ± 0.10^d^	1.15 ± 0.02^d^
C18:1n–9 c	21.69 ± 0.20^a^	16.85 ± 0.34^b^	16.64 ± 0.86^b^	15.40 ± 1.22^b^	16.48 ± 0.50^b^
C18:1n–9 t	2.38 ± 0.03^a^	1.62 ± 0.02^b^	1.31 ± 0.17^b^	1.49 ± 0.10^b^	0.46 ± 0.78^c^
C20:1n–9	0.06 ± 0.01^c^	1.01 ± 0.01^a^	0.54 ± 0.14^b^	0.52 ± 0.44^b^	0.03 ± 0.00^d^
∑MUFA	27.62 ± 0.16^a^	21.48 ± 0.40^b^	20.02 ± 0.66^b^	18.59 ± 1.67^c^	18.12 ± 0.32^c^
C18:3n–3(ALA)	0.51 ± 0.05^e^	1.41 ± 0.01^d^	2.13 ± 0.14^c^	2.63 ± 0.16^b^	3.27 ± 0.16^a^
C20:3n–3	0.02 ± 0.01^c^	0.07 ± 0.03^ab^	0.05 ± 0.00^bc^	0.07 ± 0.02^ab^	0.10 ± 0.00^a^
C20:5n–3 (EPA)	0.92 ± 0.02^a^	0.90 ± 0.02^a^	0.69 ± 0.14^b^	0.69 ± 0.11^b^	0.66 ± 0.04^b^
C22:6n–3 (DHA)	1.88 ± 0.10^b^	1.94 ± 0.22^b^	1.61 ± 0.55^c^	2.40 ± 0.33^a^	2.22 ± 0.28^ab^
∑n‐3PUFA	3.32 ± 0.07^c^	4.32 ± 0.19^bc^	4.47 ± 0.62^b^	5.78 ± 0.90^a^	6.26 ± 0.32^a^
C18:2n–6 (LA)	8.19 ± 0.20^e^	16.75 ± 0.18^d^	22.40 ± 1.02^c^	25.80 ± 0.71^b^	28.91 ± 0.60^a^
C20:4n–6 (ARA)	0.55 ± 0.05^c^	0.74 ± 0.08^b^	0.57 ± 0.21^bc^	0.88 ± 0.31^a^	0.74 ± 0.08^b^
∑n‐6PUFA	8.73 ± 0.17^e^	17.49 ± 0.05^d^	22.97 ± 0.71^c^	26.68 ± 0.40^b^	29.65 ± 0.54^a^
n‐3/n‐6 PUFA	0.38 ± 0.08^a^	0.24 ± 0.01^b^	0.19 ± 0.03^b^	0.21 ± 0.03^b^	0.21 ± 0.01^b^

*Note:* Experimental data are expressed as mean ± SD (*n* = 3). Unlike lowercase superscript letters in the same row denote statistical significance among groups (*p* < 0.05).

Abbreviations: ALA, *α*‐linolenic acid; ARA, arachidonic acid; DHA, docosahexenoic acid; EPA, eicosapentaenoic acid; LA, linoleic acid; MUFA, monounsaturated fatty acids; n‐3 PUFA, omega 3 polyunsaturated fatty acids; n‐6 PUFA, omega 6 polyunsaturated fatty acids; SFA, saturated fatty acids.

Seven essential amino acids (EAAs) and nine nonessential amino acids (NEAAs) were identified in muscle (Table 6). The top 4 most abundant AAs were, in order, glutamate, aspartate, lysine, and leucine, while histidine was present in the lowest amount (Table 6). The amounts of delicious amino acids (DAAs), total amino acids (TAAs), and EAAs were higher in the SO3, SO6, and SO9 groups, whereas their respective minimums appeared in the SO12 group (*P* < 0.05; Table 6).

**Table 6:** Amino acid profiles in the muscle of juvenile gibel carp fed SO‐based diets with different lipid contents (dry matter, %).

Items	Groups				
	SO0	SO3	SO6	SO9	SO12
Asp	8.34 ± 0.12^c^	8.89 ± 0.05^a^	8.76 ± 0.13^ab^	8.45 ± 0.23^bc^	7.77 ± 0.14^d^
Thr	3.33 ± 0.05^b^	3.54 ± 0.02^ab^	3.52 ± 0.09^ab^	3.61 ± 0.15^a^	3.40 ± 0.11^ab^
Ser	2.89 ± 0.04^bc^	3.09 ± 0.03^a^	3.04 ± 0.05^a^	3.01 ± 0.05^ab^	2.85 ± 0.05^c^
Glu	9.85 ± 0.20^b^	10.54 ± 0.07^a^	10.54 ± 0.21^a^	10.49 ± 0.11^a^	9.69 ± 0.06^b^
Gly	3.46 ± 0.06^b^	3.61 ± 0.03^a^	3.60 ± 0.06^a^	3.64 ± 0.02^a^	3.41 ± 0.01^b^
Ala	4.53 ± 0.06^c^	4.83 ± 0.02^a^	4.77 ± 0.06^ab^	4.70 ± 0.01^b^	4.38 ± 0.02^d^
Cys	3.30 ± 0.02^b^	3.49 ± 0.02^a^	3.41 ± 0.07^ab^	3.40 ± 0.08^ab^	3.14 ± 0.03^c^
Val	3.43 ± 0.08^b^	3.59 ± 0.01^a^	3.61 ± 0.09^a^	3.63 ± 0.02^a^	3.33 ± 0.03^b^
Met	3.87 ± 0.03^b^	4.05 ± 0.03^a^	4.00 ± 0.06^ab^	3.94 ± 0.10^ab^	3.69 ± 0.02^c^
Ile	3.55 ± 0.09^bc^	3.70 ± 0.01^ab^	3.74 ± 0.10^a^	3.74 ± 0.01^a^	3.41 ± 0.05^c^
Leu	6.94 ± 0.12^b^	7.35 ± 0.03^a^	7.25 ± 0.20^a^	7.27 ± 0.07^a^	6.76 ± 0.03^b^
Phe	2.75 ± 0.28	3.04 ± 0.25	2.37 ± 0.26	2.38 ± 0.29	2.80 ± 0.35
Lys	7.95 ± 0.14^b^	8.43 ± 0.02^a^	8.36 ± 0.14^a^	8.34 ± 0.03^a^	7.61 ± 0.01^c^
His	0.11 ± 0.01	0.13 ± 0.01	0.13 ± 0.02	0.12 ± 0.01	0.13 ± 0.01
Arg	4.37 ± 0.05^b^	4.60 ± 0.07^a^	4.60 ± 0.07^a^	4.56 ± 0.05^a^	4.36 ± 0.04^b^
Pro	1.99 ± 0.07	2.11 ± 0.08	2.09 ± 0.06	2.10 ± 0.09	2.03 ± 0.08
DAAs	26.17 ± 0.44^b^	27.87 ± 0.15^a^	27.67 ± 0.40^a^	27.28 ± 0.13^a^	25.24 ± 0.18^c^
TAAs	70.66 ± 0.97^b^	75.00 ± 0.68^a^	73.39 ± 0.99^a^	73.37 ± 0.54^a^	68.76 ± 0.39^b^
EAAs	36.31 ± 0.42^b^	38.44 ± 0.40^a^	37.59 ± 0.47^a^	37.58 ± 0.41^a^	35.50 ± 0.42^b^
TAAs/EAAs	51.38 ± 0.19	51.25 ± 0.08	50.94 ± 0.07	51.22 ± 0.19	51.62 ± 0.35

*Note*: Experimental data are expressed as mean ± SD (*n* = 3). Unlike lowercase superscript letters in the same row denote statistical significance among groups (*p* < 0.05).

Abbreviations: DAAs, delicious amino acids (Asp, Glu, Gly, Ala, Phe); EAAs, essential amino acids (His, Thr, Val, Met, Phe, Ile, Leu, Lys); TAAs, total amino acids.

### 3.3. Serum Biochemical Indexes

Serum ALT and AST activities were diminished to varying degrees in response to an increase in dietary SO from 0% to 9% (*p* < 0.05; Figure 1), while their levels in the SO12 group returned to near to or higher than those of the control (i.e., SO0) (*p* < 0.05; Figure 1). However, the serum HDL content first rose and then dropped, exhibiting the largest and smallest values in the SO9 and SO12 groups (*p* < 0.05; Figure 1), respectively. Both serum LDL and T‐CHO incrementally increased to their respective peaks when dietary SO inclusions were raised from 0% to 12% (*p* < 0.05; Figure 1). Serum TG was significantly elevated in the 12% dietary SO‐supplemented group (*p* < 0.05; Figure 1), despite no alteration in fish receiving 0%–9% dietary SO.

**Figure 1:**
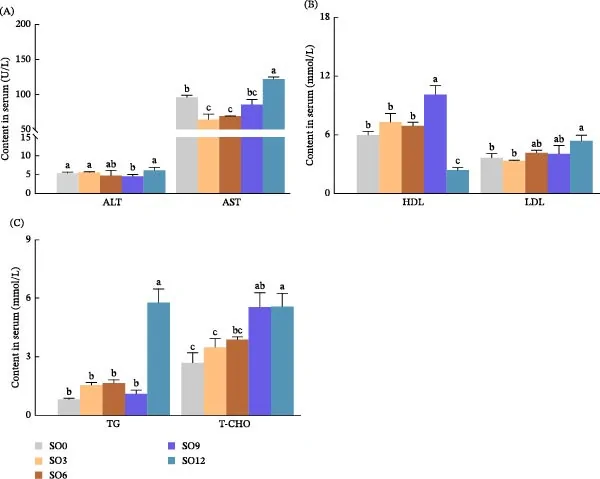
Effects of dietary SO inclusion in the SO–based diets on serum biochemical indices in juvenile gibel carp. Contents of ALT and AST (A), HDL and LDL (B), and TG and T‐CHO (C) in the serum of juvenile gibel carp fed SO–based diets with different lipid contents. Experimental data are presented as mean ± SD (*n* = 6). Unlike lowercase superscript letters in the columns denote statistical significance among groups (*p* < 0.05).

### 3.4. Hepatopancreatic Histopathological Examination

Hepatopancreatic vacuolation was observed in all treatments, and its presence increased with the contents of dietary SO addition, particularly in the SO9 and SO12 groups (Figure 2, Supporting Information 2: Figure S4). More obvious inflammatory and hemorrhage phenomena were found within the central veins and hepatic sinusoids after the supplementation of 9%–12% dietary SO (Figure 2).

**Figure 2:**
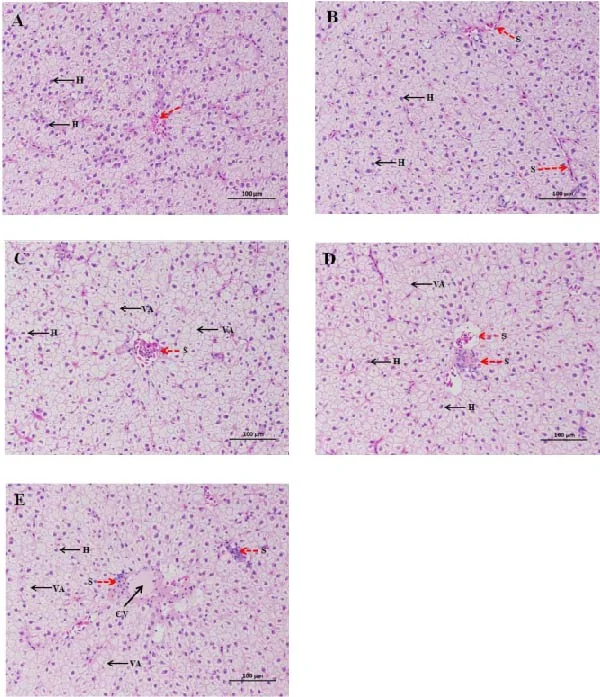
Effects of dietary SO inclusion in SO–based diets on hepatopancreatic morphology in juvenile gibel carp. Representative images of HE‐stained hepatopancreatic sections in juvenile gibel carp fed the SO0 (A), SO3 (B), SO6 (C), SO9 (D) or SO12 (E) diets. H, hepatocytes; CV, central vein; VA, vacuolization; S, sinusoids; scale bar, 100 μm.

### 3.5. Gene Expression Related to Lipid Metabolism in the Hepatopancreas

With the incremental increases in dietary SO contents, three lipogenesis‐associated genes, long‐chain acyl‐CoA synthases 1 (*acsl1*), fatty acid desaturases (*fad*), and phospholipase A2 (*pla2*), were all significantly upregulated (*p* < 0.05; Figure 3). The peak expressions of *acsl1* and *fad* were found in the SO9 group (*p* < 0.05; Figure 3), whereas maximum *pla2* occurred in the SO12 group (*p* < 0.05; Figure 3). Likewise, mRNA levels of *cd 36*, fatty acid binding protein (*fabp*), and AMP‐activated protein kinase beta 1 (*ampkβ1*) exhibited progressive upregulation as dietary SO contents increased from 0% to 12%, with significantly higher abundances in the SO9 and SO12 groups (*p* < 0.05; Figure 3). Compared with the control group, carnitine palmitoyltransferase 1 alpha (*cpt1a*) and lipoprotein lipase (*lpl*) were transcriptionally higher to variable degrees in fish fed the SO‐added diets (*p* < 0.05; Figure 3). No remarkable differences in expression of these two genes were observed among groups receiving 3%–12% dietary SO (*p* > 0.05; Figure 3), with the exception of a significant downregulation of *lpl* in the SO 12 group (*p* < 0.05; Figure 3).

**Figure 3:**
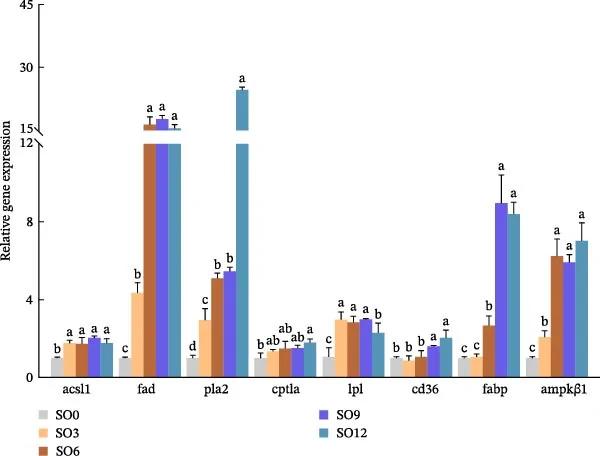
Effects of dietary SO inclusion in SO–based diets on lipid metabolism gene expression in the hepatopancreas of juvenile gibel carp. Relative abundances of lipid metabolism‐associated genes in the hepatopancreas of juvenile gibel carp fed SO–based diets with different lipid contents. Experimental data are presented as mean ± SD (*n* = 3). Unlike lowercase superscript letters in the columns denote statistical significance among groups (*p* < 0.05).

### 3.6. Detection and Enrichment Analysis of DEGs and Validation of Transcriptomic Data

Using the criterion of | log2 (fold change)| >1 and adjusted *p*  < 0.05, 4481 DEGs were identified in the comparison of SO12 vs. SO0, comprising 2576 upregulated and 1905 downregulated DEGs (Figure 4, Supporting Information 1: Table S4).

**Figure 4:**
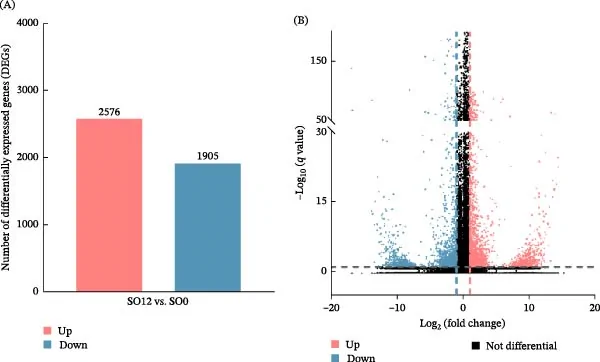
Differentially expressed genes (DEGs) in gibel carp hepatopancreas identified between the SO12 and SO0 groups. Bar graph (A) and volcano map (B) of DEGs in the comparison of SO12 vs. SO0. Pink column/dot, light blue column/dot, and black dots denote significantly upregulated DEGs, significantly downregulated DEGs, and genes without significant expression change, respectively. The criteria for screening DEGs was defined at |log2(fold change)| > 1 and adjusted *p* < 0.05.

GO functional annotation revealed 312 significantly enriched GO terms in the SO12 vs. SO0 comparison, which could be further classified into 230 terms for biological process (BP), 62 terms for cellular component (CC), and 20 GO terms for molecular function (MF) (Figure 5A, Supporting Information 3: Table S5). Among them, the significantly enriched GO terms with the highest DEG numbers in BP, CC, and MF ontologies were response to stimulus (GO:0050896), membrane part (GO:0044425), and identical protein binding (GO:0042802), respectively (Figure 5A, Supporting Information 2: Figure S6, Supporting Information 3: Table S5). Of 4481 DEGs, 658 genes were mapped to seven known pathways with significant enrichment, including steroid biosynthesis and protein digestion and absorption (Figure 5B, Supporting Information 4: Table S6). Downregulated DEGs were highly assigned to nine signaling pathways. Among them, the dominant pathways were associated with nutrient metabolism, such as the PPAR signaling pathway, fatty acid metabolism, fatty acid biosynthesis, and protein digestion and absorption (Supporting Information 2: Figure S7, Supporting Information 4: Table S6). Dramatically enriched pathways of upregulated DEGs were steroid biosynthesis, cell cycle, and so on (Supporting Information 2: Figure S7, Supporting Information 4: Table S6).

**Figure 5:**
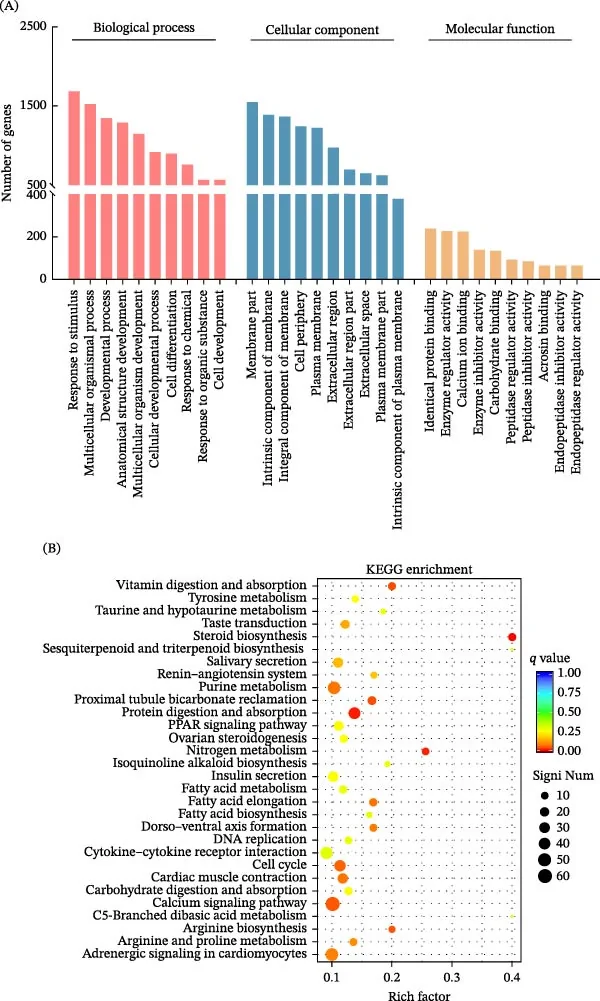
Gene ontology (GO) and Kyoto Encyclopedia of Genes and Genomes (KEGG) classification of DEGs in the hepatopancreas of gibel carp between the SO12 and SO0 groups. (A) Significantly enriched GO annotation of DEGs in the comparison of SO12 vs. SO0. X‐axis denotes the top 10 significantly enriched GO terms in the three GO ontologies. Y‐axis denotes the number of DEGs enriched in the GO term. The length and color of each column denote the number of DEGs significantly enriched in a GO term and three main GO categories, respectively. The criteria for identifying significantly enriched GO terms were defined at adjusted *p* < 0.05. (B) Significantly enriched KEGG pathway of DEGs in the comparison of SO12 vs. SO0. X‐axis denotes the rich factor. Y‐axis denotes the top 20 enriched KEGG pathway. The area and color intensity of each bubble denote the number of DEGs annotated in a KEGG pathway and the significance of enrichment in the corresponding pathway, respectively. The criteria for identifying significantly enriched KEGG pathways were defined at adjusted *p* < 0.05.

Additionally, as expected, the relative expressions of most randomly picked DEGs exhibited consistent trends with RNA‐seq data (Figure 6, Supporting Information 1: Table S7).

**Figure 6:**
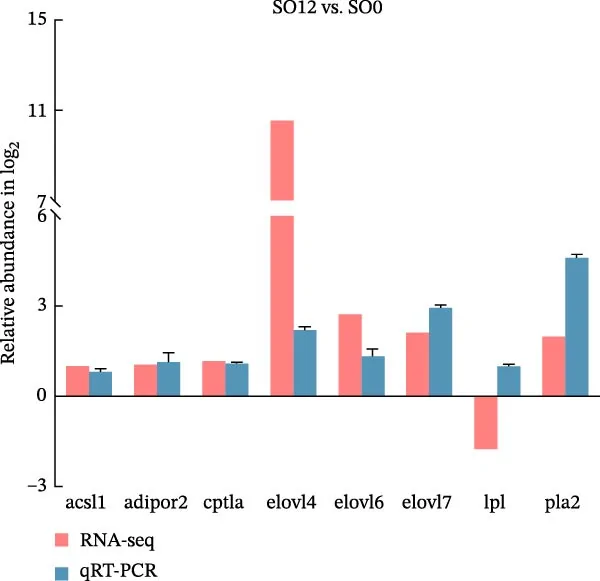
Validation of the expression of DEGs by qRT‐PCR. Relative abundances of 8 DEGs in the hepatopancreas of gibel carp between the SO12 and SO0 groups. Each gene was subjected to qRT‐PCR and normalized to the candidate reference genes (*ef1a* and *rpl13a*). Experimental data are presented as mean ± SD (*n* = 3).

## 4. Discussion

### 4.1. Effects of Different SO Levels on Growth Performance in Juvenile Gibel Carp

Concordant with previous studies [7, 29, 30], herein, VSI was continuously elevated with increasing fat contents in SO–based diets, suggesting a positive association between dietary fat intake and visceral mass. Likewise, dietary SO, serving as the main lipid source, dose‐dependently increased the FW, WGR, and SGR in gibel carp, achieving their respective maximum in fish fed diets with 12% SO supplementation (Table 3). However, no differences in these parameters, especially for WGR and SGR, were found after administration of 6%–12% dietary SO. Furthermore, based on the regression analysis of FCR (Supporting Information 2: Figure S1), 6.37% SO addition was optimal for gibel carp, considerably lower than the supplementation amount of SO estimated by the regression analysis of WGR and SGR (Supporting Information 2: Figure S2). This level falls in the recommended ranges of dietary fat concentration (4%–11%) for several cyprinoid species fed with the single SO–based diets [7, 17, 18, 31].

Noteworthily, given the finding that high levels of n‐6 PUFA‐enriched SO and other plant oils hamper growth and exacerbate fat deposition in aquatic animals [10, 26, 32–34], growth impediment and fatty liver will likely occur in gibel carp if dietary SO addition further increases. This assumption could be partly supported by the CF and serum parameters in this study. However, following administration of SO‐ or other plant oil‐based feeds, no significant alterations in growth parameters have been reported in some fish, regardless of the dietary fat level [35, 36]. These inconsistent results may be attributed to different lipid demands in different fish species of different sizes, ages, and health/nutrition statuses, as well as difference in feeding durations [7, 30, 37, 38].

### 4.2. Effects of Different SO Levels on Nutritional Composition in Juvenile Gibel Carp

A finding that crude fat content in gibel carp muscle increased with higher dietary SO inclusion was anticipated, due to a positive link between body fat content and dietary fat levels (particularly a high fat intake) observed in multiple fishes [7, 17, 19, 30, 39, 40]. This further corroborates that fat deposition in the fish body largely depends on exogenous lipid mass and dietary fatty acid profiles [41]. The increase in muscle crude protein caused by dietary SO supplementation was concentration‐specific and disappeared/reversed with high dietary SO addition (12% or more). Thus, long‐term excessive addition of dietary SO could fail to improve protein retention for more flesh production in gibel carp, as has been demonstrated in some aquatic animals fed high‐fat diets [7, 17, 18, 30, 42]. Notably, increases in dietary lipid concentrations that may trigger the aforementioned effect can differ among various fishes [19, 30, 39, 40, 43].

Unlike the consistently reduced saturated fatty acid (SFA) and monounsaturated fatty acid (MUFA) contents in gibel carp muscle, a continual rise was found for muscle n‐3 PUFAs and n‐6 PUFAs, where DHA and arachidonic acid (ARA) were maximized at 9% SO supplementation, and higher muscle LA and ALA levels appeared at 6%–12% SO addition (Table 5). Our observations indicated that DHA and ARA can be generated by converting SO‐sourced n‐6 PUFAs in gibel carp, in contrast to findings reported for seawater fish [9, 44]. In addition to the higher muscle deposition of LA, which is the main n‐6 PUFA in SO, a greater amount of DHA in gibel carp muscle was observed compared to EPA, agreeing with previous reports, including on cyprinoid and noncyprinoid fish [7, 45, 46]. This might result from a substrate‐competitive effect between n‐3 and n‐6 PUFAs and their relevant metabolites for the conversion of PUFAs, particularly LC‐PUFAs [47]. Moreover, SO‐sourced ALA (at least partial ALA) can ultimately be metabolized to DHA within the animal body [48, 49], and most freshwater fish can endogenously synthesize DHA from EPA through different enzymatic pathways [7], which may also cause increased DHA accumulations in gibel carp receiving SO–based feeds.

Free amino acid (AA) composition is another key parameter of nutritional value in flesh [28], with most AAs and total amino acids (TAAs) exhibiting significantly increased levels with the addition of 3%–9% dietary SO in this study (Table 6). A similar trend was true for umami AA (such as Glu and Asp) and sweet‐taste AA in gibel carp muscle, conferring a nicer flavor and more sweetness to the flesh [50]. Additionally, umami AA showed more prominent concentration changes induced by dietary SO inclusion than those of sweet‐tasting AA. However, up to 12% supplementation with SO prevented the increase in AA, reducing most AAs and TAAs to their minimums or near control values. This may imply that suitable SO supplementation has beneficial effects on gibel carp flesh by enhancing flavor (most evidently) and sweetness.

### 4.3. Effects of Different SO Levels on Serum Biochemical and Morphological Properties Related to Liver Health in Juvenile Gibel Carp

Excess dietary fats can be discharged into the blood, leading to a dose‐dependent elevation of TG/T‐CHO accumulation in serum, and can also impair lipoprotein synthesis/secretion or FA degradation, ultimately triggering a detrimental loop of lipotoxicity [30, 33, 51]. Substantially increased serum TG and T‐CHO levels in gibel carp receiving 12% dietary SO are, therefore, expected. Furthermore, the serum TG and T‐CHO alterations generally corresponded with the histopathological results. Besides more hepatopancreatic insults observed with high dietary SO addition, intestinal pathological damages were evident and clearly dose‐dependent in gibel carp fed SO–based diets (Supporting Information 2: Figure S5), which have been highlighted in several fishes feeding a high proportion of dietary SO [7, 17, 26, 30]. In parallel, serum AST, one sensitive indicator of hepatic damage [30], was higher in the SO12 group than those in the other groups, suggesting potential hepatic stress in gibel carp with higher dietary SO intake. These observations further indicated that an SO–based formula with 12% SO and beyond might metabolically burden the liver and other tissues in gibel carp, thereby leading to physiological and pathological changes strongly linked to metabolism disorders in fish [4, 7, 33, 52] and decreasing the quality of aquatic foods [30, 53].

Serum LDL, another parameter related to fat absorption and metabolism [2], seemed to be positively upregulated as dietary SO inclusion increased. Simultaneous elevation in serum HDL content occurred in gibel carp consuming 0%–9% dietary SO, which could partially clear serum cholesterol. However, following 12% dietary SO addition, a marked reduction in serum HDL, combined with the marked rise in serum LDL and T‐CHO to their respective peaks, implied unbalanced fat absorption, metabolism, and transport in gibel carp. Based on our data and the relevant results of other studies [21, 54], SO–based diets with 12% SO (i.e., 13.93% dietary lipid) or higher SO elicited an overabundance of TG, T‐CHO, and other fat components in the hepatopancreas of gibel carp. These compounds were unable to fully metabolize, thereby conferring a higher risk related to metabolic disease [4, 17, 30, 46].

### 4.4. Effects of Different SO Levels on Hepatopancreas Lipid Metabolism in Juvenile Gibel Carp

Expressions of fatty acid translocase/CD36 (*cd36*) and fatty acid binding protein (*fabp*), typical transport proteins in FA transport [55, 56], were positively enhanced with dietary SO concentration (Figure 3), suggesting more FA flux from extracellular and circulating fats into the gibel carp hepatopancreas for further metabolization. Correspondingly, due to the greater amounts of these substrates under high dietary SO consumption, more active lipogenic responses occurred in the hepatopancreas of gibel carp, especially long‐chain fatty acid (LCFA) biosynthesis. In the present research, this manifested in elevated expressions of fatty acid desaturases (*fad*), phospholipase A2 (*pla2*), and long‐chain acyl‐CoA synthases 1 (*acsl 1*) genes with increasing SO contents (Figure 3). An upregulation of lipogenesis genes/proteins in response to increased dietary SO and other plant oil inclusions has been demonstrated in other aquatic animals, including Atlantic salmon (*Salmo salar*) [57], grass carp [7], largemouth bass (*Micropterus salmoides*) [30], and large yellow croaker (*Larimichthys crocea*) [33].

On the other hand, a large amount of lipids and the relevant substrates derived from SO–based diets, when transported to the liver, undergo lipolysis mainly through beta‐oxidation signaling [58], leading to a gradual rise in carnitine palmitoyltransferase 1 alpha (*cpt1a*) and lipoprotein lipase (*lpl*) genes. Nevertheless, *lpl* was substantially downregulated in the SO12 group, indicating that prolonged intake of an HFD might suppress LPL synthesis/secretion in the hepatopancreas of gibel carp. Similar decreased LPL expression has been reported in multiple fishes treated with an HFD [33, 40, 46, 59], especially those with high‐dose plant oil supplementation. Noteworthily, both lipogenic and lipolytic genes in this study were transcriptionally upregulated with increasing dietary SO concentration; the former was much more upregulated than the latter. Overall, excessive lipid levels in SO–based diets likely disrupt the subtle lipid metabolism balance and lead to more fat deposition and even fat over‐accumulation in the hepatopancreas of gibel carp, in agreement with previous reports [7, 50, 60].

### 4.5. Effects of Different SO Levels on Hepatopancreas Transcriptomic Characteristics in Juvenile Gibel Carp

Given the findings of the above‐detected parameters and the detrimental effects of excessive dietary fat reported in farmed fish and fish products [19, 23, 30, 61], hepatopancreases of the SO0 and SO12 groups were used to further investigate the molecular changes in gibel carp induced by high SO contents at the transcriptome level. Biological process (BP) occupied the largest proportion within all GO categories, in which more than half of the top 10 GO terms with the most DEGs were involved in growth and development (Supporting Information 2: Figure S6, Supporting Information 3: Table S5). This was also observed in the GO analysis of upregulated DEGs, indicating that genes corresponding to these GO entries may contribute to growth in gibel carp. These data partially support the growth phenotypes described earlier, and somewhat validate the growth‐promoting effect in fishes induced by higher‐dietary‐fat supplements or short‐term HFDs [7, 23, 25, 51, 61].

Significantly enriched KEGG pathways were mainly related to nutrient and metabolism signaling, in which “steroid biosynthesis” was most prominently enriched (Figure 5B, Supporting Information 4: Table S6). Vegetable oils, including SO, are important sources of phytosterols, which are plant‐derived steroids with multiple physiological functions [62]. With higher dietary SO levels, more phytosterols are consumed by gibel carp and thus more are involved in generating steroid derivatives as substrates. Thus, there is potential potentiation of steroid synthesis response and the relevant signaling in the hepatopancreas. In addition to affecting cholesterol (CHO) transport and absorption within the body by targeting HDLs, LDLs, and related enzymes/proteins [63, 64], these phytosterols, structurally similar to CHOs, could increase TG decomposition [63], thereby mediating lipid homeostasis in gibel carp. Our data are partly supported by relevant studies of some aquatic animals [4, 20, 51, 65].

Among all DEGs confirmed by qRT‐PCR, the upregulation of *acsl1*, *pla2*, and three ELOVL family members (*elovl 4*, *elovl 6*, and *elovl 7*) (Figure 6, Supporting Information 1: Table S7) suggested that boosting synthetic activity and LCFA production leads to further fat accumulation/utilization in the hepatopancreas of gibel carp consuming high amounts of SO, which can be corroborated by the increase in serum TG and lipogenic gene expression described above. Strikingly, *lpl*, identified as a downregulated DEG, was significantly increased in fish fed with 12% SO (Figure 6, Supporting Information 1: Table S7). In gibel carp, greater hepatic CHO accumulation and phytosterol levels, induced by a high SO intake, might also evoke an increase in *lpl*; both of these require Lpl‐involved transport [4, 63]. Furthermore, *lpl* abundance was higher in the SO12 group than in the SO0 group but significantly lower than in the other groups (Figure 3), indirectly suggesting that suppression of *lpl* expression/activity could occur upon further increases in dietary SO. Based on these data, a high dose of SO in diets may affect lipid and steroid metabolism‐associated signaling, leading to modification of the lipid metabolism in gibel carp.

## 5. Conclusion

Feeds supplemented with less than 3% SO as a single fat source might fail to meet growth and metabolic demands in juvenile gibel carp, while up to 6%–9% SO inclusion, in particular of 6.37% SO concentration estimated from FCR regression, could obtain superior growth performance and flesh quality. Fish fed diets containing 12% SO exhibited less feed utilization and muscle protein deposition, higher muscle lipid accumulation and n‐6 PUFA contents, and abnormal phenotypes regarding hepatopancreas histology and serum parameters, which in turn had negative effects on flesh nutrition and fish health. Concomitantly, greater lipid build‐up in the hepatopancreas occurred through transcriptional reinforcement of genes related to multiple steps in lipid metabolism; lipid anabolic genes were much more upregulated than lipid catabolic genes. Using RNA‐seq, 4481 DEGs were identified in the hepatopancreas of SO12 group compared to the SO0 group and were predominantly enriched in the metabolic pathways linked to lipid metabolism, that is, steroid biosynthesis, etc. Overall, the recommended amount of dietary SO as the sole fat source for juvenile gibel carp was 6.37%. This amount could facilitate superior growth and fish health and lead to better‐quality fish meat.

## Author Contributions

**Xiangning Chen**: conceptualization, methodology, formal analysis, software, funding acquisition, writing and editing. **Huiqing Li**: formal analysis, investigation, validation, visualization. **Letian Qu**: investigation, data curation, formal analysis, validation. **Zheqiu Li** and **Zhiwen Chen**: data curation, resources. **Zikui Yang** and **Teng Xia**: resources. **Jianhe Xu**: project administration. **Hanliang Cheng**: conceptualization, supervision, project administration, funding acquisition, reviewing. **Qingqing Shi**: methodology, project administration, writing and reviewing and editing.

## Funding

This study is supported by the Natural Science Foundation of Jiangsu Province (Grant BK20201465), the Postgraduate Research & Practice Innovation Program of Jiangsu Province (Grants KYCX2025‐22, KYCX24_3670, and SJCX24_2086), the Technical Development Program of Shanghai ZhengZhu Biotechnology Co., Ltd. (Grant JOUH24454), the Undergraduate Innovation and Entrepreneurship Training Program of Jiangsu Province (Grants 202511641007, SZ202511641631002, and SY202611641631018), the Priority Academic Program Development of Jiangsu Higher Education Institutions (Grant 2022JSPAPD017), and the Open Foundation of the Jiangsu Key Laboratory of Marine Biotechnology from Jiangsu Ocean University (Grant HS2021003).

## Conflicts of Interest

The authors declare no conflicts of interest.

## Supporting Information

Additional supporting information can be found online in the Supporting Information section.

## Supporting information


**Supporting Information 1** Table S1: Feed formulation and nutritional compositions of the experimental feeds (dry matter, %). Table S2: Fatty acid profiles of the experimental feeds (total identified fatty acids, %). Table S3: Slope of primers used for qRT‐PCR assay. Table S4: Statistics of DEGs in the hepatopancreas of gibel carp in the SO12 vs. SO0 comparison. Table S7: Information on lipid metabolism‐related DEGs identified by RNA‐seq.

**Supporting Information 2** Figure S1: Second‐order polynomial regression analysis of feed conversion ratio (FCR) to different levels of dietary SO supplementation in juvenile gibel carp. Figure S2: Second‐order polynomial regression analysis of weight gain rate (WGR) and specific growth rate (SGR) to different levels of dietary SO supplementation in juvenile gibel carp. Figure S3: Pie chart of the main fatty acid relative proportion in the muscle of juvenile gibel carp fed SO‐based diets with different lipid contents (total identified fatty acids, %). Figure S4: Vacuolated areas of HE‐stained hepatopancreatic sections in juvenile gibel carp fed SO–based diets with different lipid contents. Figure S5: Histopathological analysis of HE‐stained intestine sections in juvenile gibel carp fed SO–based diets with different lipid contents. Figure S6: GO enrichment analysis of upregulated and downregulated DEGs in the hepatopancreas of gibel carp. Figure S7: KEGG pathway enrichment of upregulated and downregulated DEGs in the hepatopancreas of gibel carp.

**Supporting Information 3** Table S5: Statistical information on GO enrichment analysis of DEGs in the hepatopancreas of gibel carp in the SO12 vs. SO0 comparison.

**Supporting Information 4** Table S6: Statistical information on KEGG enrichment analysis of DEGs in the hepatopancreas of gibel carp in the SO12 vs. SO0 comparison.

## Data Availability

All data are presented in the main paper and the Supporting Information and available from the corresponding author upon reasonable request.
