# Supplementary material for: Comprehensive Investigation on the Influence of Dietary Soybean Oil Levels in a Soybean Oil‐Based Diet on Gibel Carp (Carassius gibelio): Insights Regarding Growth, Nutrition, and Physiological, Metabolic, and Transcriptomic Analyses
Source: Aquac Nutr. 2026 Sep 29;2026:9199711. doi: 10.1155/anu/9199711 (PMC13624541; doi:10.1155/anu/9199711)
Supplement: Supplementary file 1 [file ANU-2026-9199711-s001.docx]

**Table S1. Feed formulation and nutritional composition of the experimental feeds (dry matter, %).**

| **Items** | **Groups** | | | | |
| --- | --- | --- | --- | --- | --- |
|  | **SO0** | **SO3** | **SO6** | **SO9** | **SO12** |
| **Ingredients (%)** |  |  |  |  |  |
| Fish meal | 15.00 | 15.00 | 15.00 | 15.00 | 15.00 |
| Soybean meal | 23.00 | 23.00 | 23.00 | 23.00 | 23.00 |
| Canola meal | 11.80 | 11.80 | 11.80 | 11.80 | 11.80 |
| Cottonseed meal | 18.00 | 18.00 | 18.00 | 18.00 | 18.00 |
| Flour | 29.00 | 26.00 | 23.00 | 20.00 | 17.00 |
| Ca(H_2_PO_4_)_2_ | 1.80 | 1.80 | 1.80 | 1.80 | 1.80 |
| L-Lys | 0.15 | 0.15 | 0.15 | 0.15 | 0.15 |
| DL-Met | 0.05 | 0.05 | 0.05 | 0.05 | 0.05 |
| Choline | 0.30 | 0.30 | 0.30 | 0.30 | 0.30 |
| Soybean oil | 0.00 | 3.00 | 6.00 | 9.00 | 12.00 |
| Vitamin premix ^#^ | 0.40 | 0.40 | 0.40 | 0.40 | 0.40 |
| Mineral premix ^*^ | 0.50 | 0.50 | 0.50 | 0.50 | 0.50 |
| Total | 100 | 100 | 100 | 100 | 100 |
| **Chemical composition (%)** |  |  |  |  |  |
| Crude lipid | 2.17±0.10 | 5.11±0.23 | 8.05±0.18 | 10.99±0.26 | 13.93±0.14 |
| Crude protein | 35.24±0.32 | 35.23±0.25 | 35.19±0.18 | 35.20±0.20 | 35.18±0.19 |

Note:

^#^ Vitamin premix (each/kg diet): VE, 50 mg; VA, 5,000 IU; VD, 2000 IU; VK, 5mg; VB1, 8 mg; VB6, 8 mg; VB2, 10 mg; VB12, 0.03 mg; pantothenic acid, 30 mg; folic acid, 3 mg; nicotinic acid, 30 mg; inositol, 100 mg; biotin, 0.4 mg; VC, 180 mg.

*Mineral premix (each/kg diet): Mg, 300mg; Zn, 150 mg; Fe, 170 mg; Co, 0.25 mg; Cu, 4 mg; Mn, 22 mg; Se, 0.4 mg.

Data in Table S1 have been published in our previous report (Chen et al., 2024; DOI: 10.1016/j.aqrep.2024.101916).

**Table S2.** **Fatty acid profile of the experimental feeds (total identified fatty acids, %)**

| **Items** | **Groups** | | | | |
| --- | --- | --- | --- | --- | --- |
|  | **SO0** | **SO3** | **SO6** | **SO9** | **SO12** |
| C12:0 | 0.15±0.02^a^ | 0.06±0.00^b^ | 0.05±0.00^b^ | 0.04±0.00^b^ | 0.04±0.00^b^ |
| C14:0 | 7.62±0.09^a^ | 3.89±0.25^b^ | 3.30±0.16^c^ | 2.58±0.09^d^ | 2.21±0.10^d^ |
| C16:0 | 39.97±0.06^a^ | 34.24±1.26^b^ | 30.52±1.63^c^ | 29.30±0.54^c^ | 28.97±0.45^c^ |
| C18:0 | 8.38±0.08^b^ | 9.26±0.09^a^ | 9.59±0.69^a^ | 9.32±0.05^a^ | 9.32±0.17^a^ |
| C20:0 | - | 0.61±0.02^b^ | 0.73±0.09^a^ | 0.63±0.01^ab^ | 0.57±0.00^b^ |
| SFA | 56.11±0.12^a^ | 48.07±1.25^b^ | 44.19±1.22^c^ | 41.87±0.63^d^ | 41.11±0.44^d^ |
| C16:1 | 2.63±0.03^a^ | 1.44±0.01^b^ | 1.16±0.07^c^ | 0.91±0.01^d^ | 0.78±0.01^e^ |
| C18:1n-9 | 12.48±0.18^c^ | 13.10±0.42^c^ | 14.25±0.38^b^ | 14.88±0.39^ab^ | 15.45±0.10^a^ |
| C20:1n-9 | 1.51±0.02^a^ | 0.18±0.00^b^ | 0.16±0.00^b^ | 0.01±0.00^c^ | 0.01±0.00^c^ |
| MUFA | 16.63±0.19^a^ | 14.71±0.44^c^ | 15.57±0.31^b^ | 15.80±0.39^ab^ | 16.24±0.10^ab^ |
| C18:3n-3 (ALA) | 3.01±0.02^b^ | 3.91±0.35^b^ | 5.48±0.84^a^ | 5.65±0.11^a^ | 6.15±0.18^a^ |
| C20:5n-3 (EPA) | 3.62±0.06^a^ | 1.96±0.01^b^ | 1.51±0.04^c^ | 1.17±0.02^d^ | 0.97±0.01^e^ |
| C22:6n-3 (DHA) | 2.44±0.13^a^ | 1.29±0.03^b^ | 1.03±0.02^c^ | 0.71±0.00^d^ | 0.66±0.03^d^ |
| n-3 PUFA | 9.06±0.07^a^ | 7.16±0.35^b^ | 8.03±0.88^ab^ | 7.53±0.09^b^ | 7.78±0.15^b^ |
| C18:2n-6 (LA) | 18.19±0.11^c^ | 29.92±0.98^b^ | 32.11±2.33^ab^ | 34.73±0.91^a^ | 34.81±0.20^a^ |
| C20:4n-6 (ARA) | - | 0.12±0.00^a^ | 0.10±0.00^b^ | 0.06±0.00^c^ | 0.05±0.00^d^ |
| n-6 PUFA | 18.19±0.11^c^ | 30.05±0.98^b^ | 32.21±2.33^ab^ | 34.80±0.91^a^ | 34.87±0.20^a^ |

Note:

Experimental data are presented as mean ± SD (n=3). Unlike lowercase superscript letters in the same row denote statistical significance (*P*<0.05).

-: not detected in this study.

Abbreviations for Table S2 are as follows:

ALA: α-linolenic acid; ARA: arachidonic acid; DHA: docosahexenoic acid; EPA: eicosapentaenoic acid; LA: linoleic acid; SFA: saturated fatty acids; MUFA: monounsaturated fatty acids; n-3 PUFA: omega 3 polyunsaturated fatty acids; n-6 PUFA: omega 6 polyunsaturated fatty acids.

Data in Table S2 have been published in our previous report (Chen et al., 2024; DOI: 10.1016/j.aqrep.2024.101916).

**Table S3. Slope of primers used for qRT-PCR assay.**

| **Gene** | **Slope** |
| --- | --- |
| *acsl1* | -3.42 |
| *adipor2* | -3.26 |
| *ampkβ1* | -3.29 |
| *cd36* | -3.50 |
| *cpt1a* | -3.40 |
| *elovl4* | -3.54 |
| *elovl6* | -3.38 |
| *elovl7* | -3.49 |
| *fabp* | -3.47 |
| *fad* | -3.56 |
| *lpl* | -3.27 |
| *pla2* | -3.58 |
| *ef1a* | -3.44 |
| *rpl13a* | -3.55 |

Note:

Abbreviations of gene name in Table S3 were as follows:

*acsl1*: long-chain acyl-CoA synthases 1; *adipor2*: adiponectin receptor 2; *ampkβ1*: AMP-activated protein kinase β1; *cd36*: fatty acid translocase/CD36; *cpt1a*: carnitine palmitoyltransferase 1 alpha; *elovl4*: elongase of very long-chain fatty acids 4; *elovl6*: elongase of very long-chain fatty acids 6; *elovl7*: elongase of very long-chain fatty acids 7; *fabp*: fatty acid binding protein; *fad*: fatty acid desaturases; *lpl*: lipoprotein lipase; *pla2*: phospholipase A2; *ef1a*: elongation factor 1 alpha; *rpl13a*: ribosomal protein L13a.

**Table S4. Statistics of DEGs in the hepatopancreas of gibel carp in the SO12 *vs.* SO0 comparison.**

| **Group** | **DEGs** | | |
| --- | --- | --- | --- |
|  | **Upregulated** | **Downregulated** | **Total** |
| SO12 vs. SO0 | 2576 | 1905 | 4481 |

Note:

Hepatopancreatic samples in the control group were named as SO0 (fed on diets supplementing with 0% soybean oil (SO) as a single fat source for 12 weeks). Hepatopancreatic samples in the experimental group were named as SO12 (fed on diets supplementing with 12% SO as a single fat source for 12 weeks). Three replicates were used for each group in the transcriptome analysis.

DEGs in the comparison of SO12 *vs.* SO0 were screened at |log2(fold change)| >1 and corrected *P*<0.05.

**Table S7. Information on lipid metabolism-related DEGs identified by RNA-seq.**

| **Gene ID** | **Name** | **Mean TPM (SO12)** | **MeanTPM (SO0)** | **Log2(fold change) //corrected *P*-value** | **Gene description** |
| --- | --- | --- | --- | --- | --- |
| LOC113039684 | *acsl1* | 0.5100 | 0.2532 | 1.01↑ // 1.30E-04 | long-chain-fatty-acid--CoA ligase 1-like |
| LOC113048279 | *adipor2* | 22.8684 | 10.9304 | 1.07↑ // 2.39E-192 | adiponectin receptor protein 2-like, transcript variant X2 |
| LOC113073066 | *cpt1a* | 1.5543 | 0.6910 | 1.16↑ // 2.11E-17 | carnitine O-palmitoyltransferase 1, liver isoform-like |
| LOC113065652 | *elovl4* | 0.1492 | 0.0001 | 10.54↑ // 9.49E-07 | elongation of very long chain fatty acids protein 4-like |
| LOC113057295 | *elovl6* | 1.0448 | 0.1589 | 2.71↑ // 6.32E-27 | elongation of very long chain fatty acids protein 6-like |
| LOC113110194 | *elovl7* | 1.0454 | 0.2399 | 2.12↑// 7.39E-11 | elongation of very long chain fatty acids protein 7-like |
| LOC113040150 | *lpl* | 0.2383 | 0.8131 | -1.77 🡓 // 4.56E-11 | lipoprotein lipase-like |
| LOC113070145 | *pla2* | 0.1122 | 0.0283 | 1.98↑// 0.02 | cytosolic phospholipase A2 gamma-like |

Note:

Hepatopancreatic samples in the control group were named as SO0 (fed on diets supplementing with 0% soybean oil (SO) as a single fat source for 12 weeks). Hepatopancreatic samples in the experimental group were named as SO12 (fed on diets supplementing with 12% SO as a single fat source for 12 weeks).

Gene names (|log2(fold change)| >1 and corrected *P*<0.05) and fold changes in the transcript abundances of these genes were analyzed by RNA-seq.

Abbreviations for Table S7 are as follows:

*acsl1*: long-chain acyl-CoA synthases 1; *adipor2*: adiponectin receptor 2; *cpt1a*: carnitine palmitoyltransferase 1 alpha; *elovl4*: elongase of very long-chain fatty acids 4; *elovl6*: elongase of very long-chain fatty acids 6; *elovl7*: elongase of very long-chain fatty acids 7; *lpl*: lipoprotein lipase; *pla2*: phospholipase A2.
