# Supplementary material for: Comprehensive Investigation on the Influence of Dietary Soybean Oil Levels in a Soybean Oil‐Based Diet on Gibel Carp (Carassius gibelio): Insights Regarding Growth, Nutrition, and Physiological, Metabolic, and Transcriptomic Analyses
Source: Aquac Nutr. 2026 Sep 29;2026:9199711. doi: 10.1155/anu/9199711 (PMC13624541; doi:10.1155/anu/9199711)
Supplement: Supplementary file 2 [file ANU-2026-9199711-s002.docx]

**Fig.S1.**

**Fig.S1. Second-order polynomial regression analysis of feed conversion ratio (FCR) to different levels of dietary SO supplementation in juvenile gibel carp.**

Relationship between feed conversion ratio (FCR) and dietary SO contents used for gibel carp.

The horizontal axis denotes the value of FCR. The vertical axis denotes the different dietary SO contents (%). Each point in the regression model represents the mean value of three replicates.

The regression equation for FCR: y = 0.004206x**^2^**-0.05348x+1.326; R2 = 0.9540; Xmin=6.37.

**Fig.S2.**

**Fig.S2. Second-order polynomial regression analysis of weight gain rate (WGR) and specific growth rate (SGR) to different levels of dietary SO supplementation in juvenile gibel carp.**

(A) Relationship between weight gain rate (WGR) and dietary SO contents used for gibel carp.

(B) Relationship between specific growth rate (SGR) and dietary SO contents used for gibel carp.

The horizontal axis denotes the value of WGR (%) and SGR (%), respectively. The vertical axis denotes the different dietary SO contents (%). Each point in the regression model represents the mean value of three replicates.

The regression equation for WGR: y = -0.9400x**^2^**+20.35x+164.04; R2 = 0.9740; Xmax=10.82.

The regression equation for SGR: y = -0.003556x**^2^**+0.07780x+1.148; R2 = 0.9686; Xmax=10.82.

**Fig.S3.**

**Fig.S3. Pie chart of the main fatty acid relative proportion in the muscle of juvenile gibel carp fed SO-based diets with different lipid contents.**

Proportions of main fatty acid types in the muscle of juvenile gibel carp fed the SO0 (A), SO3 (B), SO6 (C), SO9 (D), and SO12 (E) diets.

SFA: saturated fatty acid; MUFA: monounsaturated fatty acid; n-3 PUFA: omega 3 polyunsaturated fatty acid; n-6 PUFA: omega 6 polyunsaturated fatty acid.

**Fig.S4.**

**Fig.S4.** **Vacuolated areas of HE-stained hepatopancreatic sections in juvenile gibel carp fed SO-based diets with different lipid contents.**

The area percentage of hepatopancreatic vacuolation was quantified by dividing the vacuolated area to the total area.

Experimental data are presented as mean ± SD (n = 3) and were assessed using the Welch’s ANOVA test. Unlike lowercase superscript letters in the columns denote statistical significance among groups (*P* < 0.05).

**Fig.S5.**

**Fig.S5.** **Histopathological analysis of HE-stained intestine sections in juvenile gibel carp fed SO-based diets with different lipid contents.**

Representative images of HE-stained intestine sections in juvenile gibel carp fed SO-based diets with different lipid contents.

Black arrow: exfoliated epithelial cells in the apical portion of intestinal villi; Red arrow: cytoplasmic vacuolation; Blue arrow: increased lymphocytes.

Scale bar: 1000 μm, 100 μm.

**Fig.S6.**

**Fig.S6. GO enrichment analysis of upregulated and downregulated differentially expressed genes (DEGs) in the hepatopancreas of gibel carp.**

GO classification of upregulated (A) and downregulated (B) DEGs in the comparison of SO12 *vs.* SO0.

The horizontal axis denotes the number of DEGs with a significantly enriched GO term (corrected *P* ≤ 0.05). The vertical axis represents the significantly enriched GO term. The GO terms are grouped into three categories: biological process (BP), cellular component (CC), and molecular function (MF).

SO12: Hepatopancreas in gibel carp receiving 12% dietary SO; SO0: Hepatopancreas in gibel carp receiving 0% dietary SO.

**Fig.S7.**

**Fig.S7. KEGG pathway enrichment of upregulated and downregulated differentially expressed genes (DEGs) in the hepatopancreas of gibel carp.**

Top 30 enriched KEGG pathways of upregulated (A) and downregulated (B) DEGs in the comparison of SO12 *vs.* SO0.

The horizontal axis denotes the rich factor. The vertical axis represents the name of enriched KEGG pathway (Top 30).

The area and color intensity of each bubble denote the number of DEGs annotated in a KEGG pathway and the significance of enrichment in the corresponding pathway, respectively.

SO12: Hepatopancreas in gibel carp receiving 12% dietary SO; SO0: Hepatopancreas in gibel carp receiving 0% dietary SO.
